# Fixed-Dose Artesunate–Amodiaquine Combination vs Chloroquine for Treatment of Uncomplicated Blood Stage P. vivax Infection in the Brazilian Amazon: An Open-Label Randomized, Controlled Trial

**DOI:** 10.1093/cid/ciw706

**Published:** 2016-12-16

**Authors:** Andre M. Siqueira, Aline C. Alencar, Gisely C. Melo, Belisa L. Magalhaes, Kim Machado, Aristóteles C. Alencar Filho, Andrea Kuehn, Marly M. Marques, Monica Costa Manso, Ingrid Felger, José L. F. Vieira, Valerie Lameyre, Claudio T. Daniel-Ribeiro, Marcus V. G. Lacerda

**Affiliations:** 1 ^1^ Fundação de Medicina Tropical Dr. Heitor Vieira Dourado,; 2 ^2^ Universidade do Estado do Amazonas, Manaus,; 3 ^3^ Instituto Nacional de Infectologia Evandro Chagas, Fundação Oswaldo Cruz, Rio de Janeiro,; 4 ^4^ Universidade Federal do Amazonas, Manaus, Brazil;; 5 ^5^ ISGlobal, Barcelona Center for International Health Research (CRESIB), Hospital Clínic - Universitat de Barcelona, Barcelona, Spain;; 6 ^6^ Swiss Tropical and Public Health Institute, Basel, Switzerland;; 7 ^7^ Universidade Federal do Pará, Belém, Brazil;; 8 ^8^ Access to Medicines Department, Sanofi , Paris, France;; 9 ^9^ Instituto Oswaldo Cruz, Fundação Oswaldo Cruz, Rio de Janeiro, and; 10 ^10^ Instituto Leônidas e Maria Deane, Fundação Oswaldo Cruz, Manaus, Brazil

**Keywords:** *Plasmodium vivax*, malaria, chloroquine, randomized clinical trial, artesunate-amodiaquine.

## Abstract

**Background.:**

Despite increasing evidence of the development of *Plasmodium vivax* chloroquine (CQ) resistance, there have been no trials comparing its efficacy with that of artemisinin-based combination therapies (ACTs) in Latin America.

**Methods.:**

This randomized controlled trial compared the antischizontocidal efficacy and safety of a 3-day supervised treatment of the fixed-dose combination artesunate-amodiaquine Winthrop^®^ (ASAQ) versus CQ for treatment of uncomplicated *P. vivax* infection in Manaus, Brazil. Patients were followed for 42 days. Primary endpoints were adequate clinical and parasitological responses (ACPR) rates at day 28. Genotype-adjustment was performed.

**Results.:**

From 2012 to 2013, 380 patients were enrolled. In the per-protocol (PP) analysis, adjusted-ACPR was achieved in 100% (165/165) and 93.6% (161/172) of patients in the ASAQ and CQ arm (difference 6.4%, 95% CI 2.7%; 10.1%) at day 28 and in 97.4% (151/155) and 77.7% (129/166), respectively (difference 19.7%, 95% CI 12.9%; 26.5%), at day 42. Apart from ITT D28 assessment, superiority of ASAQ on ACPR was demonstrated. ASAQ presented faster clearance of parasitaemia and fever. Based on CQ blood level measurements, CQ resistance prevalence was estimated at 11.5% (95% CI: 7.5-17.3) up to day 42. At least one emergent adverse event (AE) was recorded for 79/190 (41x6%) in the ASAQ group and for 85/190 (44x7%) in the CQ group. Both treatments had similar safety profiles.

**Conclusions.:**

ASAQ exhibited high efficacy against CQ resistant P. vivax and is an adequate alternative in the study area. Studies with an efficacious comparator, longer follow-up and genotype-adjustment can improve CQR characterization.

**Clinical Trials Registration**. NCT01378286.

## Summary

In the Brazilian Amazon, the artesunate–amodiaquine combination was more effective in preventing *Plasmodium vivax* recurrence. With a favorable safety profile, this antimalarial treatment proved to be a good first-line alternative. Chloroquine resistance is probably underestimated in the area.

There were an estimated 214 million malaria episodes and 438 000 related deaths in 2015 [1]. *Plasmodium vivax* is the malaria-causing parasite species with the widest geographical distribution, resulting in 2.85 billion people at risk [2]. Studies from all *P. vivax* endemic areas demonstrate that this infection can progress to have severe and fatal outcomes [3, 4], rendering the long-held belief that *P. vivax* is benign as no longer valid [5].

Resistance to antimalarials is a major barrier for case-management and control of transmission [6, 7] and requires constant monitoring. For more than 60 years, the mainstay of *P. vivax* treatment has been a combination of chloroquine (CQ) with primaquine (PQ) as an antirelapse drug [8, 9]. This combination’s synergistic schizontocidal effect, which is associated with the parasite’s usually lower biomass and shorter duration of gametocytes presence compared with those of *Plasmodium falciparum*, may have contributed to slower development of CQ resistance in *P. vivax* [9]. However, there has been an increasing number of reports of CQ resistance (CQR) in regions where this parasite is endemic [10–12], especially in Southeast Asia and the Pacific region [13–15], where 5 countries have already adopted artemisinin-based combination therapies (ACTs). these therapies provide fast parasite clearance and prevent recrudescences [16] as first-line treatment for *P. vivax* [1]. In Latin America, where *P. vivax* is the most prevalent species causing malaria [1] despite recent evidence of CQR [11, 17–20], no studies comparing the efficacy of ACTs to that of CQ have been reported.

The combination of artesunate–amodiaquine (ASAQ) against *P. falciparum* has been adopted in many countries and is one of the World Health Organization (WHO) prequalified antimalarials. The ASAQ fixed-dose combination (FDC) tested in this study (artesunate–amodiaquine Winthrop, or ASAQ Winthrop) was codeveloped by DNDi and Sanofi. This formulation was shown to result in better compliance and reduced risk of emergence of *P. falciparum* resistance when compared with loose-dose combinations and coblisters [21, 22]. We conducted this clinical trial in order to evaluate the efficacy and safety of the ASAQ FDC compared with that of CQ against uncomplicated *P. vivax* infection.

## METHODS

### Study Site

Our study was undertaken at Fundação de Medicina Tropical Dr. Heitor Vieira Dourado (FMT-HVD), Manaus, in the Brazilian Western Amazon region. Malaria transmission is restricted to rural areas, and *P. vivax* causes around 90% of malaria cases [23]. Antimalarials are only provided in health units after diagnostic confirmation of infection. National guidelines recommend CQ (total dose of 25 mg/kg over 3 days) and PQ (0.5 mg/kg/day over 7 days) for *P. vivax* infection treatment and artemether–lumefantrine or artesunate–mefloquine for *P. falciparum* [24]. Studies showed therapeutic failure rates as high as 10% with CQ monotherapy for *P. vivax* in 2005 [17].

### Study Design

This was an open-label, randomized, noninferiority, controlled trial that compared the efficacy and safety of ASAQ (Winthrop; Sanofi, Morocco) and CQ (Farmanguinhos, Brazil) for treatment of uncomplicated *P. vivax* blood stage infection. The study was based on the WHO antimalarial drug efficacy protocols modified for *P. vivax* [25, 26]. Patients were followed up for 42 days to assess drug efficacy and safety.

The ethics review board of FMT-HVD (0426/2011) and the National Brazilian Committee of Ethics (16532/2011) approved the study. All adult patients and legal guardians of children provided written informed consent. The protocol is available in the Supplementary Materials.

### Patients

Individuals aged >6 months with a body weight >5 kg with slide-confirmed *P. vivax* monoinfection, parasite density between 250 and 100 000 parasites/µL, and axillary temperature ≥37.5°C or history of fever in the last 48 hours were considered eligible. Exclusion criteria included pregnant or breast-feeding women; plan of leaving the study area in the following 42 days; known hypersensitivity to 1 of the investigational products; blurred vision suggesting retinopathy; presence of at least 1 severe criterion of malaria; known severe concomitant or underlying disease; and women of childbearing potential unwilling to use an effective contraceptive method during the study.

### Procedures, Randomization, and Concealment

Enrolled patients were randomly assigned to 1 of the treatment arms by a study nurse through a computer-generated list that linked each patient’s study number to an opaque zone, thus, concealing the assigned treatment arm. Neither the study doctors nor the laboratory personnel responsible for reading the slides and the polymerase chain reaction (PCR) test had access to patients’ allocation during the study.

ASAQ was administered as a fixed-dose combination with 3 oral formulations (25/67.5 mg, 50/135 mg, 100/270 mg) and CQ as 150-mg tablets. Dosing was based on established weight bands for each drug (Supplementary Table 1). Study nurses supervised all dosing at enrollment and 24 and 48 hours after. When necessary, tablets were dissolved in water and administered orally using a syringe to ensure proper dosing to children. Patients were followed on an outpatient basis. PQ was withheld until day 42 or at day of recurrence. Recurrences during follow-up were treated with fixed-dose artesunate–mefloquine combination (Farmanguinhos, Brazil). The dose was administered a second time if vomiting occurred within 30 minutes.

Visits for clinical and microscopic assessments were scheduled on days 1, 2, 3, 7, 14, 28, and 42 after enrollment. Laboratory blood assessments (total blood cell count, bilirubin, glycemia, aspartate aminotransferase, alanine aminotransferase [ALT], and creatinine) were performed at enrollment and repeated on day 7 and day 28. Electrocardiograms were performed at enrollment, day 2, and day 28 for all participants aged >10 years and evaluated by a cardiologist. Blood samples were collected on day 7 and day of recurrence for CQ and desetylchloroquine (DCQ) blood assays, measured using high-performance liquid chromatography as previously described [27]. The sum of CQ and DCQ blood levels was used to classify parasites as sensitive or resistance based on the 100-ng/mL cutoff level, as previously established [12, 26, 28]. Technical problems with sample storage and collection, consisting of insufficient volume of blood for the analyses, prevented measurement of day 7 blood levels of AQ.

Two experienced technicians examined microscopic blood smears. Parasite density was calculated using the white cell count from the nearest total blood cell count (usually from enrollment, day 7, or day 28) [29].

### Molecular Analyses

Real-time PCR was performed on filter-paper samples from enrollment or recurrences to confirm *P. vivax* monoinfection according to described protocols [30]. For the genotyping procedures for comparing day 0 and recurrence samples, 3 highly polymorphic gene regions were chosen according to diversity and discriminatory potential in the region, namely, msp1F3, MS2, and MS8 [31–34]. As there is no standard recommendation for classifying *P. vivax* recurrences due to difficulties distinguishing reinfection, recrudescence, and relapse [35, 36], we adapted the classification method recommended by WHO for *P. falciparum* [25]. For each recurrence, samples were classified as homologous if at least 1 allele for each loci investigated was detected in both paired samples and as heterologous if all alleles for a given marker were different. A recurrent homologous *P. vivax* episode could derive either from a recrudescent-resistant parasite or from a dormant liver stage. For this study, we chose to treat these events as treatment failures after correction. Further details on the molecular characterization are provided in the Supplementary Materials.

### Statistical Analyses and Sample Calculation

We assumed a 90% efficacy rate for CQ within 28 days [17] and 95% efficacy for ASAQ [37]. The noninferiority margin was defined at 5%, considering alpha at 2.5% for the unilateral test and power of 90%, a sample size of 145 individuals per arm, increased to 190 after adding 15% loss to follow-up and 10% risk of mixed infections.

The primary endpoint was genotype-adjusted adequate cure and parasitological response (ACPR) at day 28 for the per-protocol (PP) population. Secondary endpoints, performed in both the PP and intention-to-treat (ITT) populations, included crude ACPR on day 28 and ACPR on day 14 and day 42, prevalence and incidence of positive asexual parasitemia and gametocyte carriage, hemoglobin changes from baseline, and incidence of adverse events (AEs).

Noninferiority was demonstrated if the lower bound of the 95% confidence interval (CI) of the risk difference between the 2 arms was greater than −0.05 in the PP population populations. The CIs were calculated using normal approximation, with continuity correction in the Wald limits applied in the case of empty cells [38]. If noninferiority was shown, a 2-sided Fisher exact test to assess superiority was performed in the ITT population. This analysis was performed despite not being described in the protocol-following recommendation [39] and due to the large difference observed. Both the PP and ITT are presented.

Time to recurrence was assessed by survival curves using the Kaplan-Meier estimator and log-rank tests. The proportion of patients with negative blood slides for both asexual and sexual parasites and fever clearance was analyzed at days 1, 2, and 3. A general estimating equation (GEE) model [40] was used to compare person-week gametocyte densities during follow-up; this consisted of a univariate analysis with robust standard errors, with the number of gametocyte-positive slides per individual per week as the outcome.

Anemia was graded according to WHO references [41]. Neutropenia and elevation of ALT were considered AEs of special interest [42–44]. An independent data monitoring committee comprised of 3 renowned experts reviewed the study protocol and evaluated the results of efficacy and safety analyses. An additional analysis was performed only for the CQ arm to investigate a possible association between delayed parasite clearance (>72 hours) as the dependent variable and risk of recurrence, the outcome variable, using logistic regression.

## RESULTS

**Figure 1.**
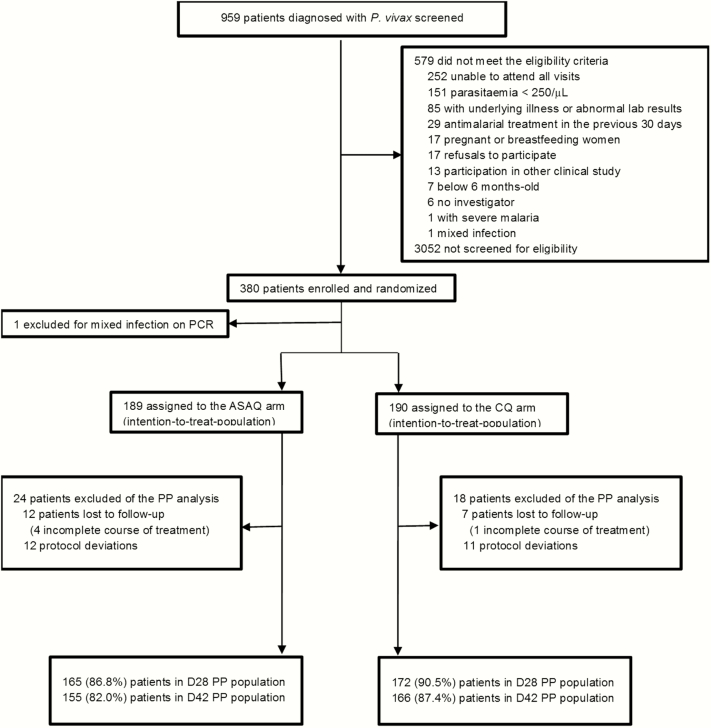
Flowchart of the study. *Some patients had more than 1 reason for exclusion. Abbreviations: ASAQ, artesunate–amodiaquine; CQ, chloroquine; PCR, polymerase chain reaction; PP, per protocol; *P. vivax*, *Plasmodium vivax*.

Patients were enrolled between January 2012 and June 2013 (Figure 1). Most patients were adult males and resided in the nonactive transmission Manaus urban area (Table 1).

**Table 1.** Demographic and Disease Characteristics at Baseline in the Intention-to-Treat Population

Variable	Artesunate–Amodiaquine (n = 189)	Chloroquine (n = 190)
Male	136 (72.0%)	144 (75.8%)
Age, y	35.7 (1–68; 16·4)	34.7 (1–74; 15·9)
<6	7 (3.7%)	7 (3.7%)
6–14	10 (5.3%)	14 (7.4%)
≥14	172 (91.0%)	169 (88.9%)
Weight, kg	68.8 (8.5–117.9; 18.8)	68.4 (9.5–111.0; 20.3)
Concomitant illness	25 (13.2%)	28 (14.7%)
Residing in Manaus urban area	170 (89.5%)	174 (91.6%)
Temperature (°C)	37.2 (34.9–40.1; 1.3)	37.1 (35.0–40.0; 1.3)
Temperature ≥37.5°C	85 (45.0%)	77 (40.5%)
Geometric mean parasite density per μL (95% confidence interval)	1746 (1475–2068)	1643 (1412–1912)
Positive gametocytemia	105 (55.6%)	114 (60.0%)
Hemoglobin (g/dL)	13.3 (7.3–18.3; 1.9)	13.2 (7.8–17.3; 1.7)
Anemia	93 (48.9%)	83 (43.7%)

Data are expressed as percentage or mean (range; standard deviation), unless specified otherwise.

**Figure 2.**
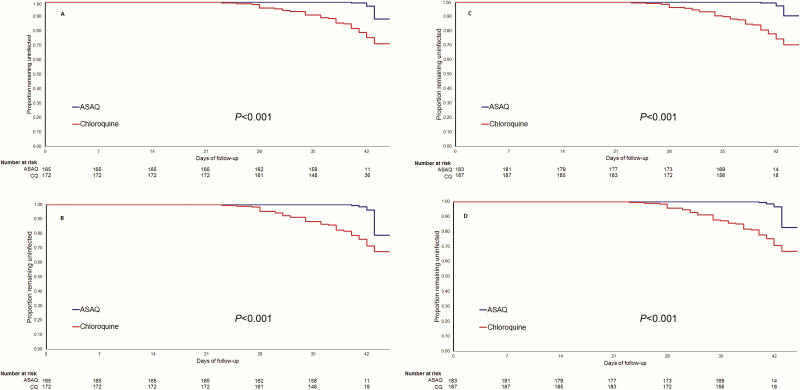
Kaplan-Meier estimates for time to recurrence. *A,* Polymerase chain reaction (PCR)–corrected per-protocol (PP) population. *B,* Crude estimate of PP population. *C,* PCR-corrected intention-to-treat (ITT) population. *D,* Crude estimate of ITT population. *P* value from log-rank test. Abbreviations: ASAQ, artesunate–amodiaquine; CQ, chloroquine.

Noninferiority was demonstrated (97.5% CI lower bound = 2.74) as genotype-adjusted ACPR rates at day 28 of 100% (165/165) for ASAQ and 93.6% (161/172) for CQ. Although superiority of ASAQ was not demonstrated on day 28 in the ITT population (*P* = .345), it was demonstrated in the PP population and on day 42 for both populations (Tables 2 and 3; Figure 2). The difference was larger between day 28 and day 42.

**Table 2.** Adequate Clinical and Parasitological Response (ACPR) by Time Point for the Per-Protocol Population

Variable	Artesunate– Amodiaquine, n = 165 (%)	Chloroquine, n = 172 (%)	Difference (95% Confidence Interval)	*P* value
**Day 28**				
Genotype-adjusted ACPR	165 (100)	161 (93.6)	6⋅4% (2⋅7–10⋅1)	⋅001
Total failures	0	11 (6.4)		
Late clinical failure	0	4 (2.3)		
Late parasitological failure	0	7 (4.1)		
*Genotype classification of failures*				
Homologous	0	10 (5.8)		
Heterologous	0	2 (1.2)		
Indeterminate	0	1 (0⋅6)		
Unadjusted ACPR	165 (100.0)	159 (92.4)	7⋅6% (3⋅6–11⋅5)	<⋅001
Total failures	0	13 (7.5)		
Late clinical failure	0	4 (2.3)		
Late parasitological failure	0	9 (5.2%		
**Day 42**	n = 155	n = 166		
Genotype-adjusted ACPR	151 (97.4)	129 (77.7)	19⋅7% (12⋅9–26⋅5)	<⋅001
Total failures	4 (2.6)	37 (22.3)		
Late clinical failure	2 (1.3)	14 (8.4)		
Late parasitological failure	2 (1.3)	23 (13.9)		
*Genotype classification of failures* ^a^				
Homologous	3(1.9)	31 (20.1)		
Heterologous	2 (1.3)	7 (4.2)		
Indeterminate polymerase chain reaction	1 (0.7)	6 (3.6)		
Unadjusted ACPR	149 (96.1)	122 (73.5)	22⋅6% (15⋅3–30⋅0)	<0⋅001
Total failure	6 (3.9)	44 (36.9)		
Late clinical failure	2 (1.3)	17 (10.2)		
Late parasitological failure	4 (2.6)	27 (16.3)		
Parasite and fever clearance				
Parasite cleared at day 1	33 (20.0)	4 (2.3)		<.001
Parasite cleared at day 2	144 (87.3)	77 (44.8)		<.001
Parasite cleared at day 3	164 (99.4)	138 (80.2)		<.001
Fever cleared at day 1	164 (99.4)	154 (89.5)		<.001

Abbreviation: ACPR, adequate cure and parasitological response.

^a^Genotyping detailed data available in Table Supplementary S1.

**Table 3.** Efficacy Assessments at Day 28 and Day 42 for the Intention-to-Treat Population

Variables	Artesunate– Amodiaquine, n = 189 (%)	Chloroquine, n = 190 (%)	Difference (95% Confidence Interval)	*P* value
**Day 28**				
Genotype-adjusted ACPR	177 (93.7)	172 (90.5)	3.1% (−2⋅3–8⋅5)	.345
Total failures	12 (6.3)	18 (9.5)	
Late clinical failure	0 (0.0)	4 (2.1)	
Late parasitological failure	0 (0.0)	7 (3.7)	
Missing data ^**a**^	12 (6.3)	7 (3.7)	
*Genotype classification of failures*				
Homologous	0	10 (5.8)	
Heterologous	0	2 (1.2)	
Indeterminate	0	1 (0.6)	
Unadjusted ACPR	177 (93.7)	170 (89.5)	4.2% (−1.4–9.8)	.201
Total failures^a^	12 (6.3)	20 (10.5)	
Late clinical failure	0	4 (2.1)	
Late parasitological failure	0	9 (4.7)	
**Day 42**				
Genotype-adjusted ACPR	162 (85.7)	136 (71.6)	14.1% (6.0–22.3)	.001
Total failures	27 (14.3)	54 (28.4)	
Late clinical failure	2 (1.1)	15 (7.9)	
Late parasitological failure	2 (1.1)	26 (13.7)	
Missing data ^**a**^	23 (12.2)	13 (6.8)	
*Genotype classification of failures*			
Homologous	3 (1.6)	34 (18.4)	
Heterologous	2 (1.1)	7 (3.7)	
Indeterminate polymerase chain reaction	1 (0.5)	7 (3.7)	
Unadjusted ACPR	160 (84.7)	129 (67.9)	16.8% (8.4–25.2)	<.001
Total failures	29 (15.5)	61 (32.1)	
Late clinical failure	2 (1.1)	18 (9.5)	
Late parasitological failure	4 (2.1)	30 (15.8)		
Parasite and fever clearance				
Parasite cleared at day 1	38 (20.1)	9 (4.7)		<.001
Parasite cleared at day 2	162 (85.7)	89 (46.8)		<.001
Parasite cleared at day 3	184 (97.4)	153 (80.5)		<.001
Fever cleared at day 1	184 (97.4)	168 (88.4)		<.001

Genotyping detailed data available in Supplementary Table S1.

Abbreviation: ACPR, adequate cure and parasitological response.

^**a**^Losses to follow-up and samples with genotype assessment..

**Figure 3.**
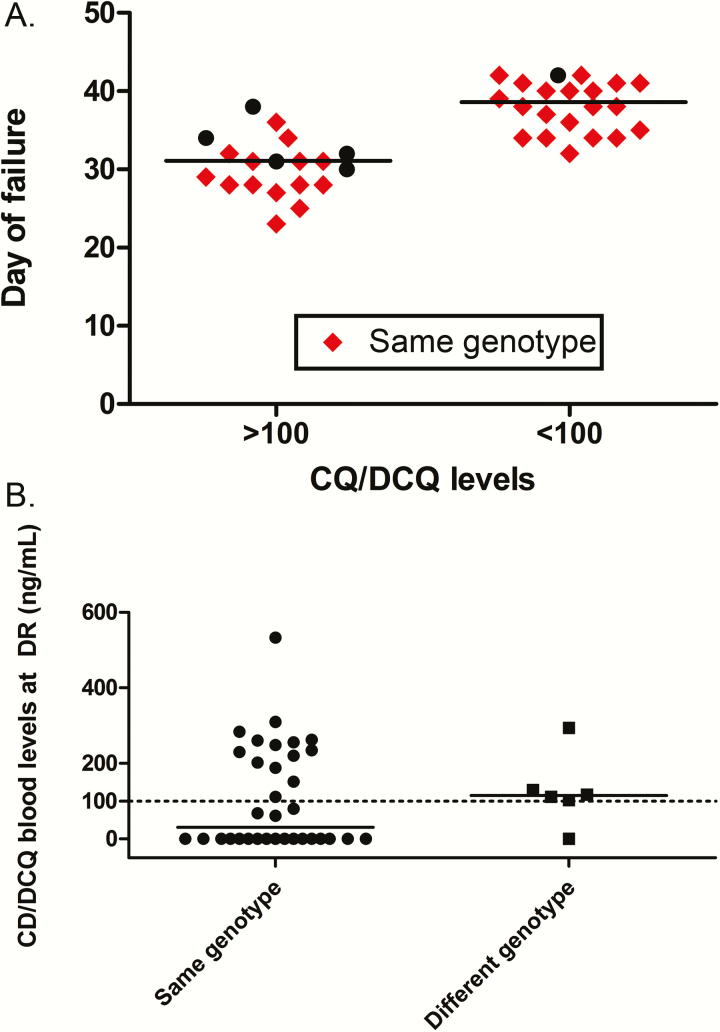
*A,* Genotype of failures in both arms is shown as same (red diamonds) or distinct (black dots) genotype according to day of follow-up (lines show the median day of failure). *B,* Chloroquine (CQ)/desetylchloroquine blood levels on the day of failure from patients in the CQ arm according to genotype classification (line demarks the 100 ng/mL threshold). Abbreviations: CQ, chloroquine; DCQ, desetylchloroquine; DR: day of reccurrence.

Through genotype classification, 64.8% (34/54) of the CQ arm recurrences (ITT population) were homologous compared with half (3/6) in the ASAQ arm (Figure 3A and Table 3). CQ/DCQ blood levels on the day of recurrence were measured for 48 of 54 patients, with 19 being >100 ng/mL, resulting in a CQR prevalence of 11.5% (95% CI, 7.5–17.3). This result was further corroborated by both homologous (14/34) and heterologous (5/6) recurrences emerging in the circulation, with CQ/DCQ blood levels as high as 533 ng/mL (Figure 3B).

Parasitemia clearance was faster, and the proportion of patients clearing fever at day 1 was higher for ASAQ (Tables 2 and 3). From day 7, 2.4% of patients treated with ASAQ and 14.7% in the CQ group presented with microscopically detected gametocytes during follow-up (*P* < .001). The GEE model demonstrated a lower overall gametocyte-positive density in the ASAQ arm compared with that of CQ (4.7 vs 12.2 gametocyte-positive slides/100 person-weeks, respectively; *P* < .001). Risk of developing gametocytemia after treatment initiation in patients with no gametocytes at inclusion (ASAQ, n = 83; CQ, n = 76) was higher for CQ (20.2% vs 55.4%; *P* < 0·001). There was no association between delayed parasite clearance and risk of recurrence (odds ratio = 1.1; 95% CI, 0.7–1.6; *P* = .836) in the CQ arm.

There was a relative decrease of hemoglobin (Hb) between inclusion and day 7 for all patients (−2.5; standard deviation = 8.4), with greater reduction observed for ASAQ (−4.2 [7.4] vs −0.9 [9.0]; *P* < .001). There was a similar proportion of patients who presented with grade 2 or higher anemia at day 7 (3 [1.6%] vs 2 [1.1%]). At day 28, patients who received CQ presented with a higher increase from baseline (0.3 [13.2] vs 3.7 [12.0]; *P* = .005). Overall AE rates were similar (Table 4). Severe AEs were only observed in the ASAQ arm, none with sequelae.

**Table 4.** Most Frequent Adverse Events Reported in the Safety Population

Variables	Artesunate– Amodiaquine, n = 190 (%)^a^	Chloroquine, n = 190 (%)
All AEs		
Patients with at least 1 event	79 (41.6)	85 (44.7)
Cardiac disorders	35 (18.4)	21 (11.1)
Skin disorders	14 (7.4)	23 (12.1)
Infections and infestations	16 (8.4)	21 (11.1)
Gastrointestinal disorders	16 (8.4)	15 (7.9)
Psychiatric disorders/insomnia	8 (4.2)	12 (6.3)
Drug-related AEs^b^		
Patients with at least 1 of the following:	59 (31.1)	47 (14.7)
Sinus bradycardia	28 (14.7)	11 (5.8)
Pruritus	13 (6.8)	19 (10.0)
Increased ALAT	9 (4.7)	8 (4.2)
Gastritis	8 (4.2)	5 (2.6)
Vomiting	6 (3.2)	5 (2.6)
Insomnia	2 (1.1)	4 (2.1)
Diarrhea	2 (1.1)	0 (0.0)
At least 1 event of special interest	8 (4.2)	9 (4.7)
Increased ALAT^c^	8 (4.2)	8 (4.2)
Neutropenia^d^	0	1 (0.5)
Serious AEs¶		
Patients presenting at least 1 serious AE*	3 (1.6)	0
Vomiting	3	0
Severe gastritis	1	0
Extrapyramidal syndrome	1	0

Only AEs that affected at least 4% in each group are listed.

Abbreviations: AE, adverse event; ALAT, alanine aminotransferase.

^**a**^ The patient with mixed infection detected by polymerase chain reaction was included in the safety analyses.

^b^ Only events that affected at least 1% of the population in each group are listed.

^c^ ALAT >5 × upper limit of normal (ULN) value, or ALAT >3 × ULN if ALAT was >ULN on day 0, or ALAT >3 × ULN associated with total bilirubin >2×ULN.

^d^ Neutrophil count <400/mm^3^ in children aged 3 months to 12 years or <750/mm^3^ in children aged >12 years or adults. ^¶^Events were classified as serious AEs based on the need for intravenous medication to alleviate the symptoms.

*There were no investigational product discontinuation or deaths in the study. Full recovery occurred for all AEs.

## DISCUSSION

We observed high efficacy of the ASAQ FDC against CQ-resistant *P. vivax* with respect to ACPR and other efficacy outcomes. This is the first study to compare the ASAQ combination with chloroquine against *P. vivax* infection and the first trial to compare an ACT with CQ in Latin America [1].

A remarkable result of this study is the high efficacy of ASAQ at day 42 (for which all recurrences occurred after day 40). Interpretation of failures occurring after day 28 is complicated by the possibility of recurrences being relapses or reinfections [9, 25]. Despite this caveat, we believe the observed late recurrences indicate the existence of low-grade resistance to CQ, which would suppress but not completely eliminate circulating parasites [12]. This is based on 2 important assumptions: very low risk of reinfection, as most patients (90.5%) resided in urban areas with no active malaria transmission [23], and negligible contribution of relapses, which usually occur after day 35 in the region [32, 45, 46].

In the absence of PQ administration, the drug’s elimination half-life is an important property to be considered against *P. vivax* due to the post-treatment prophylactic effect [9]. Its influence is illustrated by 2 trials that compared the ASAQ loose-dose combination to dihydroartemisinin–piperaquine, an ACT with a longer half-life. Lower efficacy against late recurrences of ASAQ [43] was not confirmed when PQ was coadministered [44]. This rationale is supported by the higher efficacy of CQ in preventing late recurrences compared with drugs with shorter half-lives (arthemeter–lumefantrine) in areas of low prevalence of CQR [47]. Thus, our results provide strong evidence of a higher-than-expected CQR prevalence because, even though CQ and its metabolites’ half-lives are longer, there was a large number of recurrences in the CQ arm.

The genotype-adjusted ACPR rates were measured in an attempt to gain a more rigorous case definition of CQR. Genotyping has been applied in *P. vivax* efficacy studies with interesting results [35, 36]. However, unlike in *P. falciparum*, there are no well-defined criteria to differentiate recrudescence from reinfection and relapse [25]. PCR correction will not necessarily lead to more precise estimation of efficacy in areas of low endemicity, where homologous hypnozoites are more likely [34].

ASAQ was more effective in clearing patent gametocytemia. CQ may take up to 4 days for gametocyte clearance [48]. Studies have found an association between prolonged gametocytemia and asexual stage recurrence [49]. It is not clear if use of an ACT has an effect on reducing transmission.

Hb reduction at day 7 was higher in patients who received ASAQ. Possible explanations include higher inflammatory response and/or oxidative stress associated with treatment, suppression of the blood marrow by artesunate, and pitting [50, 51]. The risk of severe anemia after ACT is considered low [52]; indeed, there was no severe anemia in our study.

The safety and tolerability of ASAQ FDC were consistent with what was found in previous studies that used this combination in the approved indication “treatment of uncomplicated *P. falciparum* malaria” [42]. Sinus bradycardia occurred without symptoms or clinical complications, and there was no difference regarding the corrected QT inverval (QTc).

A limitation of our study was the small number of children included. This population is at higher risk of treatment failure [53, 54], for which age-stratified sampling would be important. There was a higher loss to follow-up in the ASAQ arm, which had an impact on the efficacy assessment in the ITT population. An examination of the notification database did not reveal a higher rate of failure among losses. As we were not able to assess the pharmacokinetic profile of amodiaquine, evaluation of the effect of a longer elimination half-life in this population on efficacy against late recurrences was not possible.

By not coadministering PQ, we were able to estimate CQR, which at 11.5%, considering concurrent blood drug levels criterion, is very high. This is important, as patients receiving CQ are at higher risk of recurring episodes from resistant parasites that arise from either recrudescence or relapse. These findings also suggest that the usual methods for measuring CQ efficacy are prone to underestimate the true CQR rates. Most studies of *P. vivax* efficacy restrict follow-up to 28 days in order to minimize the contribution of relapses, considering CQ elimination half-life. We believe this can result in underestimation of resistance, which, in our study, could be as high as 30% based on the risk difference against an efficacious comparator. This difference highlights the need to develop better designs and strategies for measuring antimalarial efficacy against *P. vivax*.

## CONCLUSIONS

This is the first trial to compare an ACT with CQ as blood schizontocidal against *P. vivax* in Latin America. We demonstrated that CQR is underestimated in the region and that a change in treatment policy should be considered. Recently *Plasmodium vivax* has received increased attention, requiring development of new strategies and tools [55]. The choice of an effective schizontocidal drug, therefore, cannot be underestimated. Although CQ is still the first-line therapy in most endemic countries, resistance is a growing problem, probably underestimated because of coadministration with PQ and misclassification of recrudescence as relapse. The decision to substitute CQ for an ACT as the drug of choice for treating *P. vivax* infections is complex and should be based on different factors, including the prevalence of CQ-resistant parasites; the current treatment adopted for *P. falciparum*; efficacy of the partner drug; interaction of the ACT with PQ; and the potential impact of reducing transmission [13]. In order to establish unified treatment recommendations for *P. falciparum* and *P. vivax*, it is necessary to provide a more effective option and reduce the risk of treating *P. falciparum* with CQ due to misdiagnosis [50]. We believe that CQ should be replaced by a more efficacious alternative in this region and expect policymakers to consider these findings in order to define the best strategies to accelerate the path toward malaria eradication.

## Supplementary Data

Supplementary materials are available at *Clinical Infectious Diseases* online. Consisting of data provided by the author to benefit the reader, the posted materials are not copyedited and are the sole responsibility of the author, so questions or comments should be addressed to the author.

## Notes

***Funding***. This study was funded by Sanofi. The study sponsor participated in the development the protocol, interpreted the data, helped write the report, and performed monitoring.

***Acknowledgements***. We acknowledge Director-President of FMT-HVD Professor Maria Alecrim for the institutional support, all the personnel from the Malaria Laboratory for their contribution in running the trial, and the study monitor from Sanofi. We thank the patients and their families for taking part in the study. C. T. D. R. and M. V. G. L. are recipients of a grant from the Conselho Nacional de Desenvolvimento Científico e Tecnológico as *Bolsistas de Produtividade* and A. M. S. receives a short-term training grant from WHO’s Special Programme for Research and Training in Tropical Diseases.

***Author contributions***. A. M. S., V. L., C. D. T. R., and M. V. G. L. designed and coordinated the study. A. M. S., A. C. A., B. L. M., K. M., M. M. M., and M. C. M. participated in data collection. G. C. M., A. K., M. M. M., I. F., and J. L. F. V. participated in the laboratory analyses. A. M. S., V. L., and M. V. G. L. conducted the statistical analyses. A. M. S. wrote the first draft of the manuscript. All authors participated in data interpretation and approved the final version and take responsibility for accuracy and completeness of data reporting and for the decision to submit for publication.

***Potential conflicts of interest***. V. L. is employed by Sanofi. All other authors report no conflicts. All authors have submitted the ICMJE Form for Disclosure of Potential Conflicts of Interest. Conflicts that the editors consider relevant to the content of the manuscript have been disclosed.

## Supplementary Material

Supplementary Data
