# Supplementary material for: Fixed-Dose Artesunate–Amodiaquine Combination vs Chloroquine for Treatment of Uncomplicated Blood Stage P. vivax Infection in the Brazilian Amazon: An Open-Label Randomized, Controlled Trial
Source: Clin Infect Dis. 2016 Dec 16;64(2):166–74. doi: 10.1093/cid/ciw706 (PMC5215218; doi:10.1093/cid/ciw706)
Supplement: Supplementary Data [file supp_64_2_166__index.html]

Fixed-Dose Artesunate–Amodiaquine Combination vs Chloroquine for Treatment of Uncomplicated Blood Stage P. vivax Infection in the Brazilian Amazon: An Open-Label Randomized, Controlled Trial — Supplementary Data 

# Fixed-Dose Artesunate–Amodiaquine Combination vs Chloroquine for Treatment of Uncomplicated Blood Stage P. vivax Infection in the Brazilian Amazon: An Open-Label Randomized, Controlled Trial

## Supplementary Data

Data files

- Supplementary Data - Supplementary Data
- Supplementary Data - Supplementary Data
