# Supplementary material for: Fixed-Dose Artesunate–Amodiaquine Combination vs Chloroquine for Treatment of Uncomplicated Blood Stage P. vivax Infection in the Brazilian Amazon: An Open-Label Randomized, Controlled Trial
Source: Clin Infect Dis. 2016 Dec 16;64(2):166–74. doi: 10.1093/cid/ciw706 (PMC5215218; doi:10.1093/cid/ciw706)
Supplement: Supplementary Data [file supp_ciw706_Supp_table_1_v2.docx]

**Table 1. Treatment administration according to bodyweight**

| **Drug formulation** | **Body weight** | **Daily dose*** |
| --- | --- | --- |
| **Artesunate (AS) – Amodiaquine (AQ) arm** | | |
| Tablet: AS 25mg/AQ 67·5mg | ≥5 and <9 kg | 1 tablet/day |
| Tablet: AS 50mg/AQ 135mg | ≥9 and <18 kg | 1 tablet/day |
| Tablet: AS 100mg/AQ 270mg | ≥18 and <36 kg | 1 tablet/day |
| Tablet: AS 100mg/AQ 270mg | ≥36 kg | 2 tablets/day |
| **Chloroquine (CQ)** | | |
| Tablet: 150mg | ≥5 and <10 kg | day 1= ½ tablet; day 2 = ¼ tablet; day 3 = ¼ tablet |
| Tablet: 150mg | ≥10 and <15 kg | day 1= 1 tablet; day 2 = ½ tablet; day 3 = ½ tablet |
| Tablet: 150mg | ≥15 and <25 kg | day 1= 1 tablet; day 2 = 1 tablet; day 3 = 1 tablet |
| Tablet: 150mg | ≥25 and <35 kg | day 1= 2 tablets; day 2 = 2 tablets; day 3 = 2 tablets |
| Tablet: 150mg | ≥35 and <50 kg: | day 1= 3 tablets; day 2 = 2 tablets; day 3 = 2 tablets |
| Tablet: 150mg | ≥50 kg | tablets; day 2 = 3 tablets; day 3 = 3 tablets |
| *In the need to administer a fraction of a tablet, it would be crushed and dissolved in 10mL of water and the appropriate fraction was administered (i.e. ½ tablet: 5mL; ¼ tablet: 2·5mL). | | |
