# Supplementary material for: Fixed-Dose Artesunate–Amodiaquine Combination vs Chloroquine for Treatment of Uncomplicated Blood Stage P. vivax Infection in the Brazilian Amazon: An Open-Label Randomized, Controlled Trial
Source: Clin Infect Dis. 2016 Dec 16;64(2):166–74. doi: 10.1093/cid/ciw706 (PMC5215218; doi:10.1093/cid/ciw706)
Supplement: Supplementary Data [file supp_ciw706_Supplement_Text_1.docx]

**Supplement Text 1. Methods and results of the molecular characterization and classification of samples from patients presenting recurrence of parasitaemia.**

**DNA Extraction**

Genomic DNA was extracted from filter paper The extraction of total DNA from whole blood was performed using the QIAamp DNA Blood Mini Kit (Qiagen®, USA), according to the manufacturer’s protocol.

**PCR analyses for genotyping**

46 paired samples (D0, sample before treatment and DR, sample at reappearance) were analysed regarding size polymorphisms of genetic markers. The microsatellite markers MS2 and MS8 had been shown high genetic diversity in South America previously [1–5]. Msp1F3, a highly polymorphic region of the merozoite surface protein 1 was including due to reduced occurrence of PCR artefacts of this marker [6].

Nested or semi-nested PCR was carried out as described by Koepfli et al. [7] with small modifications. Primary PCR reaction was performed as a multiplex reaction, nested PCR was carried out as simplex reactions. PCR analyses were repeated up to two times if after the first run no PCR product was obtained. The primary PCR reaction volume was 15 µL containing 1.5 µL of Buffer B (Solis BioDyne), 2 mmol/L MgCl_2_, 0.3 mmol/L dNTPs (supplier), 0.27 µmol/L of each primer and 3.75 U of Taq DNA polymerase (Firepol [Solis BioDyne]). 1 µL of genomic DNA was used as template. In nested PCR reactions 0.2 mmol/L dNTPs were used, 0.25 µmol/L of each primer, 1.125 U of DNA polymerase and 1 µL of primary PCR product as template. Cycling conditions for primary PCR were as follows: initial denaturation for 1 min at 95°C followed by 30 cycles of 30 sec at 95°C, 45 sec at 59°C and 1 min at 72°C. Final elongation was for 5 min at 72°C. For each experiment at least two negative controls containing dH_2_O instead of water were included. Nested PCR was carried out at the same cycling conditions with the exception of only 25 cycles being carried out. For repetitions, reaction volume was increased to 50 µL for primary PCR and 25 µL for nested PCR, DNA template was increased to 3 - 3.5 µL and number of cycles was increased to 35 in nested PCR. PCR products were stored at 4ºC in the dark until being analysed.

**Capillary electrophoresis and data interpretation**

Successful amplification of region of interest was tested by 2 % agarose gel electrophoresis. PCR products were diluted by adding 5 µL of mQH_2_O and samples were sent to Macrogen Inc. (South Korea) for capillary electrophoresis. As size standard 500-LIZ was applied. Data were analysed with GeneMarker version 2.4.2 (SoftGenetics). Electrophoretic profiles were evulated visually for each sample with regard to amplicon length and relative abundance (peak height). Peaks below 150 bp were not considered and the minimal cut-off value for peak height was set to 500 relative fluorescence units (rfu) in order to exclude peaks resulting from background noise. So called “stuttering” (“shadow”) peaks [8] were not considered.

Capillary electrophoresis results of both samples of each pair (D0 and DR) were compared with GeneMarker software. Alleles of paired sampled were classified as equal for a maximum difference of allele size of 1 bp. According to WHO guidelines a sample was classified as recrudescence or relapse if at least one allele for each loci investigated was detected in both paired samples [9, 10]. The guidelines classify post- treatment occurring parasitemia as new infection (reinfection) if all alleles for one marker gene are distinct between D0 and DR. A new infection detected for only one locus investigated was therefore classified as “new infection” as the overall outcome [9].

1. Rezende AM, Fontes CJF, Souza JM, Couto ADA, Carvalho LH: **Microsatellite loci : determining the genetic variability of Plasmodium vivax**. 2010, **15**:718–726.

2. Marín-Menéndez A, Bardají A, Martínez-Espinosa FE, Bôtto-Menezes C, Lacerda M V, Ortiz J, Cisteró P, Piqueras M, Felger I, Müeller I, Ordi J, del Portillo H, Menéndez C, Wahlgren M, Mayor A: **Rosetting in Plasmodium vivax: a cytoadhesion phenotype associated with anaemia.** *PLoS Negl Trop Dis* 2013, **7**:e2155.

3. De Araujo FCF, de Rezende AM, Fontes CJF, Carvalho LH, Alves de Brito CF: **Multiple-clone activation of hypnozoites is the leading cause of relapse in Plasmodium vivax infection.** *PLoS One* 2012, **7**:e49871.

4. Orjuela-Sánchez P, da Silva NS, da Silva-Nunes M, Ferreira MU: **Recurrent parasitemias and population dynamics of Plasmodium vivax polymorphisms in rural Amazonia.** *Am J Trop Med Hyg* 2009, **81**:961–8.

5. Chenet SM, Schneider K a, Villegas L, Escalante A a: **Local population structure of Plasmodium: impact on malaria control and elimination.** *Malar J* 2012, **11**:412.

6. Koepfli C, Ross A, Kiniboro B, Smith TA, Zimmerman PA, Mueller I, Felger I: **Multiplicity and diversity of Plasmodium vivax infections in a highly endemic region in Papua New Guinea**. *PLoS Negl Trop Dis* 2011, **5**:1–7.

7. Koepfli C, Mueller I, Marfurt J, Goroti M, Sie A, Oa O, Genton B, Beck H-P, Felger I: **Evaluation of Plasmodium vivax genotyping markers for molecular monitoring in clinical trials.** *J Infect Dis* 2009, **199**:1074–1080.

8. Oda S, Oki E, Maehara Y, Sugimachi K: **Precise assessment of microsatellite instability using high resolution fluorescent microsatellite analysis.** *Nucleic Acids Res* 1997, **25**:3415–20.

9. WHO: **METHODS AND TECHNIQUES FOR CLINICAL TRIALS ON ANTIMALARIAL DRUG EFFICACY : genotyping to identify parasite populations**. *WHO, Geneva* 2008.

10. Barnadas C, Koepfli C, Karunajeewa H a, Siba PM, Davis TME, Mueller I: **Characterization of treatment failure in efficacy trials of drugs against Plasmodium vivax by genotyping neutral and drug resistance-associated markers.** *Antimicrob Agents Chemother* 2011, **55**:4479–81.

**Table S2. Results of the PCR genotyping procedures and classification of parasites presenting at recurrences according to the baseline genotypes.**

|  |  | **Msp1F3** | | **MS2** | | **MS8 (bp)** | | **Outcome all markers** |
| --- | --- | --- | --- | --- | --- | --- | --- | --- |
| **SampleID** | **Individual/time point** | **Amplicon Length (bp)** | **Outcome** | **Amplicon Length (bp)** | **Outcome** | **Amplicon Length (bp)** | **Outcome** |  |
| 002_D0 | 1 D0 | 271 | Re/Re | 216 | Re/Re | 235 | Re/Re | **Re/Re** |
| 002_DR | 1 DR | 271 |  | 216 |  | 235 |  |  |
| 005_D0 | 2 D0 | 230/298 | Re/Re | 193/213 | Re/Re | n/a | no result | **Re/Re** |
| 005_DR | 2 DR | 230 |  | 193 |  | 293 |  |  |
| 014_D0 | 3 D0 | 271 | Re/Re | 197 | Re/Re | 235 | Re/Re | **Re/Re** |
| 014_DR | 3 DR | 271 |  | 197 |  | 235 |  |  |
| 015_D0 | 4 D0 | 230/271 | Re/Re | 193/216 | Re/Re | 235/293 | Re/Re | **Re/Re** |
| 015_DR | 4 DR | 230/271 |  | 193/216 |  | 235/293 |  |  |
| 043_D0 | 5 D0 | 263 | Re/Re | 193 | Re/Re | 224 | no result | **Re/Re** |
| 043_DR | 5 DR | 230/263 |  | 193 |  | n/a |  |  |
| 065_D0 | 6 D0 | 230 | Re/Re | 193 | Re/Re | 293 | Re/Re | **Re/Re** |
| 065_DR | 6 DR | 230 |  | 193 |  | 293 |  |  |
| 099_D0 | 7 D0 | 271 | Re/Re | 228 | Re/Re | 287 | no result | **Re/Re** |
| 099_DR | 7 DR | 271 |  | 228 |  | n/a |  |  |
| 105_D0 | 8 D0 | 230 | Re/Re | 193 | Re/Re | 293 | Re/Re | **Re/Re** |
| 105_DR | 8 DR | 230 |  | 193 |  | 293 |  |  |
| 106_D0 | 9 D0 | 230/271 | Re/Re | 193 | Re/Re | 224 | Re/Re | **Re/Re** |
| 106_DR | 9 DR | 230 |  | 193 |  | 224 |  |  |
| 114_D0 | 10 D0 | 230 | Re/Re | 193 | Re/Re | 293/232 | Re/Re | **Re/Re** |
| 114_DR | 10 DR | 230 |  | 193 |  | 293 |  |  |
| 118_D0 | 11 D0 | 230/271 | Re/Re | 193 | New infection | n/a | no result | **New infection** |
| 118_DR | 11 DR | 230 |  | 212 |  | 259 |  |  |
| 135_D0 | 12 D0 | 230 | no result | 267 | no result | 259 | no result | **no result** |
| 135_DR | 12 DR | n/a |  | n/a |  | n/a |  |  |
| 137_D0 | 13 D0 | 230 | Re/Re | 193/212 | Re/Re | 285/293 | no result | **Re/Re** |
| 137_DR | 13 DR | 230 |  | 193 |  | n/a |  |  |
| 149_D0 | 14 D0 | 272 | Re/Re | 260/263 | Re/Re | 296 | Re/Re | **Re/Re** |
| 149_DR | 14 DR | 272 |  | 263 |  | 296 |  |  |
| 151_D0 | 15 D0 | 230 | Re/Re | 213 | New infection | n/a | no result | **New infection** |
| 151_DR | 15 DR | 230/299 |  | 162/197 |  | n/a |  |  |
| 157_D0 | 16 D0 | 272 | Re/Re | 213/263 | Re/Re | n/a | no result | **Re/Re** |
| 157_DR | 16 DR | 272 |  | 263 |  | 296 |  |  |
| 159_D0 | 17 D0 | 230/280 | Re/Re | 201 | Re/Re | 224 | no result | **Re/Re** |
| 159_DR | 17 DR | 181/230 |  | 202/209/213 |  | n/a |  |  |
| 169_D0 | 18 D0 | 272 | Re/Re | 224 | Re/Re | 218 | Re/Re | **Re/Re** |
| 169_DR | 18 DR | 272 |  | 224 |  | 218/244 |  |  |
| 178_D0 | 19 D0 | 230/271 | Re/Re | 193 | New infection | 235/293 | New infection | **New infection** |
| 178_DR | 19 DR | 272 |  | 263 |  | 296 |  |  |
| 184_D0 | 20 D0 | 271 | Re/Re | 216 | Re/Re | 235 | Re/Re | **Re/Re** |
| 184_DR | 20 DR | 271 |  | 173/216 |  | 235/262/288 |  |  |
| 185_D0 | 21 D0 | 271 | Re/Re | 216 | Re/Re | 235 | Re/Re | **Re/Re** |
| 185_DR | 21 DR | 271 |  | 216 |  | 235 |  |  |
| 186_D0 | 22 D0 | 271 | Re/Re | 216 | Re/Re | n/a | no result | **Re/Re** |
| 186_DR | 22 DR | 271 |  | 217 |  | n/a |  |  |
| 193_D0 | 23 D0 | 230 | Re/Re | 197 | Re/Re | 293 | Re/Re | **Re/Re** |
| 193_DR | 23 DR | 230 |  | 197 |  | 293 |  |  |
| 206_D0 | 24 D0 | 230 | Re/Re | 212 | Re/Re | 258 | Re/Re | **Re/Re** |
| 206_DR | 24 DR | 230 |  | 212 |  | 258 |  |  |
| 207_D0 | 25 D0 | 230/271 | Re/Re | 197 | Re/Re | 235 | Re/Re | **Re/Re** |
| 207_DR | 25 DR | 271 |  | 197 |  | 235 |  |  |
| 209_D0 | 26 D0 | 272 | New infection | 205/263 | New infection | 296 | no result | **New infection** |
| 209_DR | 26 DR | 182/230 |  | 209/213 |  | n/a |  |  |
| 211_D0 | 27 D0 | 271 | New infection | 194/263 | Re/Re | n/a | no result | **New infection** |
| 211_DR | 27 DR | 230 |  | 194 |  | n/a |  |  |
| 213_D0 | 28 D0 | 217/224/230 | Re/Re | 193 | Re/Re | n/a | no result | **Re/Re** |
| 213_DR | 28 DR | 230 |  | 193 |  | 293 |  |  |
| 227_D0 | 29 D0 | 230/272 | Re/Re | 197/228/263 | New infection | 235/296 | Re/Re | **New infection** |
| 227_DR | 29 DR | 230 |  | 212 |  | 235 |  |  |
| 233_D0 | 30 D0 | 272 | Re/Re | 197/260 | Re/Re | 296 | New infection | **New infection** |
| 233_DR | 30 DR | 272 |  | 225/260 |  | 218 |  |  |
| 238_D0 | 31 D0 | 230/272 | Re/Re | 212/267/276 | Re/Re | n/a | no results | **Re/Re** |
| 238_DR | 31 DR | 230 |  | 276 |  | 225/258 |  |  |
| 246_D0 | 32 D0 | 263/313 | Re/Re | 193 | Re/Re | 224 | Re/Re | **Re/Re** |
| 246_DR | 32 DR | 263/271 |  | 193 |  | 224/250 |  |  |
| 248_D0 | 33 D0 | 230 | Re/Re | 209 | Re/Re | 299 | Re/Re | **Re/Re** |
| 248_DR | 33 DR | 230 |  | 209 |  | 299 |  |  |
| 249_D0 | 34 D0 | 230 | Re/Re | 193 | Re/Re | 293 | Re/Re | **Re/Re** |
| 249_DR | 34 DR | 230 |  | 193 |  | 293 |  |  |
| 265_D0 | 35 D0 | 230 | Re/Re | 202 | Re/Re | 224 | Re/Re | **Re/Re** |
| 265_DR | 35 DR | 230 |  | 202 |  | 224 |  |  |
| 267_D0 | 36 D0 | 272 | Re/Re | 259 | Re/Re | 296 | Re/Re | **Re/Re** |
| 267_DR | 36 DR | 272 |  | 259 |  | 296 |  |  |
| 276_D0 | 37 D0 | 230/276/280 | Re/Re | 212 | Re/Re | 235 | Re/Re | **Re/Re** |
| 276_DR | 37 DR | 230/273/280 |  | 212 |  | 235 |  |  |
| 282_D0 | 38 D0 | 273/299 | Re/Re | 197 | Re/Re | 218 | Re/Re | **Re/Re** |
| 282_DR | 38 DR | 299 |  | 197 |  | 218 |  |  |
| 283_D0 | 39 D0 | 298 | Re/Re | 197 | Re/Re | 218 | New infection | **New infection** |
| 283_DR | 39 DR | 298 |  | 197 |  | 235 |  |  |
| 285_D0 | 40 D0 | 272 | Re/Re | 197 | Re/Re | 236 | Re/Re | **Re/Re** |
| 285_DR | 40 DR | 272 |  | 197 |  | 235 |  |  |
| 295_D0 | 41 D0 | 230 | Re/Re | 271/275 | no result | 259 | no result | **Re/Re** |
| 295_DR | 41 DR | 230/271 |  | 213/275/279 |  | n/a |  |  |
| 314_D0 | 42 D0 | n/a | no result | n/a | no result | n/a | no result | **no result** |
| 314_DR | 42 DR | 230/270 |  | 213 |  | 258 |  |  |
| 322_D0 | 43 D0 | 230 | Re/Re | 213 | Re/Re | 236 | Re/Re | **Re/Re** |
| 322_DR | 43 DR | 230/272 |  | 213 |  | 236 |  |  |
| 331_D0 | 44 D0 | 272 | Re/Re | 240 | Re/Re | 302 | no result | **Re/Re** |
| 331_DR | 44 DR | 272 |  | 240 |  | n/a |  |  |
| 335_D0 | 45 D0 | 257 | Re/Re | 208 | Re/Re | 273 | Re/Re | **Re/Re** |
| 335_DR | 45 DR | 257 |  | 208 |  | 273 |  |  |
| 343_D0 | 46 D0 | 230 | Re/Re | 194 | Re/Re | 296 | no result | **Re/Re** |
| 343_DR | 46 DR | 230/272 |  | 194 |  | n/a |  |  |
| 349_D0 | 47 D0 | 230 | Re/Re | 201 | Re/Re | 287 | Re/Re | **Re/Re** |
| 349_DR | 47 DR | 230 |  | 201 |  | 288 |  |  |
| 371_D0 | 48 D0 | 263/268 | New infection | 209 | Re/Re | 255 | New infection | **New infection** |
| 371_DR | 48 DR | 181/257/230/272 |  | 119/213/209 |  | 294 |  |  |
| n/a - no PCR product obtained  Re/RE - relapse or recrudescence | | | | | | | | |
